# Incorporating a monetary variable into the Schelling model addresses the issue of a decreasing entropy trace

**DOI:** 10.1038/s41598-020-74125-6

**Published:** 2020-10-12

**Authors:** Alexander V. Mantzaris

**Affiliations:** Department of Statistics and Data Science, University of Central Florida, Orlando, 32816 USA

**Keywords:** Statistical physics, Phase transitions and critical phenomena, Computational science

## Abstract

The Schelling model of segregation has been shown to have a simulation trace which decreases the entropy of its states as the aggregate number of residential agents surrounded by a threshold of equally labeled agents increases. This introduces a paradox which goes against the second law of thermodynamics that states how entropy must increase. In the efforts to bring principles of physics into the modeling of sociological phenomena this must be addressed. A modification of the model is introduced where a monetary variable is provided to the residential agents (sampled from reported income data), and a dynamic which acts upon this variable when an agent changes its location on the grid. The entropy of the simulation over the iterations is estimated in terms of the aggregate residential homogeneity and the aggregate income homogeneity. The dynamic on the monetary variable shows that it can increase the entropy of the states over the simulation. The path of the traces with both variables in the results show that the shape of the region of entropy is followed supporting that the decrease of entropy due to the residential clustering has a parallel and independent effect increasing the entropy via the monetary variable.

## Introduction

The Schelling model of racial segregation introduced in^1,2^ has provided a simple yet flexible model for simulating the dynamics of a complex process for residential movements based upon identity^3^. The motivation of the model starts with an examination of rules and conditions which facilitate, an initially randomly ordered set of labeled agents (e.g. white and black), to produce a segregated and clustered set of agents. The basic rule set for the agents is that there is a constraint upon each agent to have a minimum number of agents with the same label in their adjacent surroundings for them to remain in the sample grid cell, or else they move to a different cell in which the sufficient number of same labelled agents exist adjacently. Each iteration of the simulation cycles through each agent to satisfy this criteria/constraint. It is interesting to note that the rule set is not complex since the local agent homogeneity criteria on the local environment has no explicit description for the macroscopic grid state, and yet it results in the whole grid state to be altered in a small number of iterations. For this reason it can spark interest from researchers not working directly on the social issues of segregation (and related issues such as polarization) to apply their domain expertise to understand the dynamics of self-organization^4^ to a greater depth just as the Conway Game of Life^5^ has provided much insight^6^. An implementation of the Schelling model for practical applications can be found in^7^ which provides an interface with visualizations.

Segregation based upon race is a continuing issue in the USA^8^ since the time of proposing the Schelling model to explain the dynamics for the emergence. There are other factors as well which can contribute or act independently such as segregation based upon a general socioeconomic status as presented in^9^ which looks at European cities and^10^ that examines Latin American cities. The separating label which residents can use to differentiate themselves can be placed upon football team support as shown in the Glasgow (UK) history of sectarianism between teams Celtic and Rangers^11^. The work of^12^ looks at some of the real world complex patterns displayed by segregation upon ethnic and religious statuses in which a utility (e.g. ability to afford moving costs) variable is integrated into a Schelling model without infringing upon the original formulation. An exploration of the monetary variable’s role in spatial heterogeneity data in urban regions for use in the Schelling model is also posed in the work of^13^ and how family income can explain the variance observed. The income data may not provide a complete representation for the more general monetary effects such as ’wealth’^14^ but provides related measure of which data is more easily accessible.

**Figure 1:**
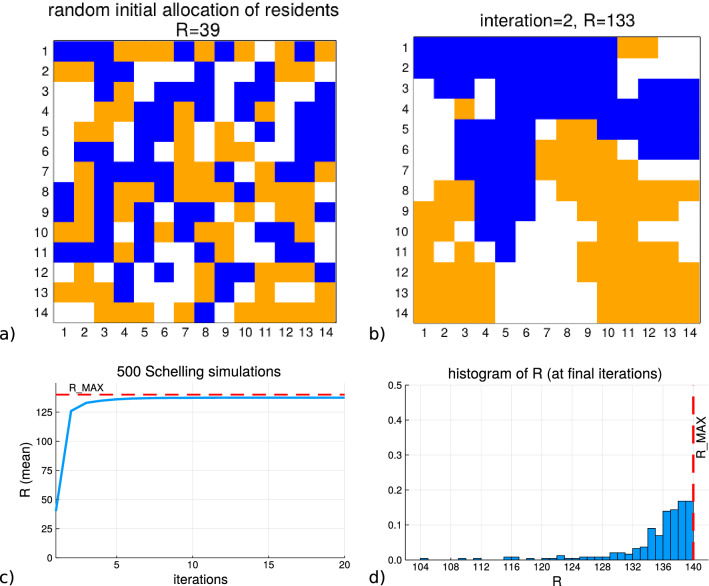
The plots demonstrate the operation of the Schelling model’s operation. **(a)** represents the first iteration of the Schelling model which starts with 2 sets of residential agents randomly placed upon a grid of empty cells. The number of agents which have their local homogeneity satisfied is $R=39$. **(b)** shows the state of the grid after a single iteration of the simulation where the number of agents with local homogeneity is $R=133$. **(c)** shows the mean value of the homogeneity satisfaction, *R*, across all the agents in the grid for each iteration from 500 simulations. **(d)** shows the distribution of the final homogeneity value *R* at each of the simulations run for **(c)**.

Figure 1 provides a set of plots in order to develop a mental image of the Schelling model’s operation. Subfigure (a) presents a random initialization of the grid where 140 agents each of 2 types (blue and orange) are randomly placed in unique cell positions. These agents are also referred to as ’residents’ and in the title of a) $R=39$ refers to the number of agents which have the number of required homogeneous residents in their adjacent grid cells in order to be ’satisfied’ under the Schelling criteria/constraint. Subfigure (b) displays the state of the grid after each resident has been given the opportunity to move to a new cell in which the homogeneity satisfaction can be achieved (one iteration forward in the simulation from the initialization). This altered the value of *R* to increase to the value of $R=133$ and the increased clustering between homogeneous labels shows this. Subfigure (c) shows the result of 500 independent simulations of the Schelling simulation with the x-axis being the iteration number and the y-axis the average *R* value across those simulation iteration values. The dashed line is the maximum possible *R* achievable for that many agents. Subfigure (d) shows the normalized histogram of the final value of *R* for those 500 simulations and it can be noticed that the simulation frequently achieves in producing configurations with *R* close to the maximum value. It is noteworthy that the Schelling model can be expected to produce these results with few iterations.

There has been continuous development of the Schelling model’s formulation in which many variants exist but the work of^15^ provides a framework in which parameterizations allow the model to capture the dynamics of a wide range of different effects within the Schelling model. A different perspective is that taken by sociophyics^16–18^ in which many tools developed for physical systems find applications in the social context with an appropriate abstraction and generalization. In^19^ a commentary about sociophysics in general is followed by the introduction of the Ising model’s^20^ formulation of the ferromagnetic interactions on the state of the spins $\{-1,+1\}$ where the aggregate affects the external magnetic field; $H(\sigma ) = -\sum _{\langle i\,j\rangle } \sigma _i \sigma _j$ and how this can relate to different human label interactions. The work of^21^ considers not only the basic locality criteria of the homogeneity satisfaction in the Schelling model as a modeling similarity with the Ising model, but also the phase changes of the system where ’spontaneous magnetism’ acts as a display of self-organising segregation. A study that investigates the phase transition behaviors under different conditions is found in^22^. The development of a ’physical analogue’ of the Schelling model is proposed in^23^ where the spatial interactions of the neighborhood of each agent is modeled with liquid tensions providing enhanced granularity to the simulations. Reference^24^ presents a probabilistic description of the model dynamics and the attractors of the configurations with the energies different states contain.

**Figure 2:**
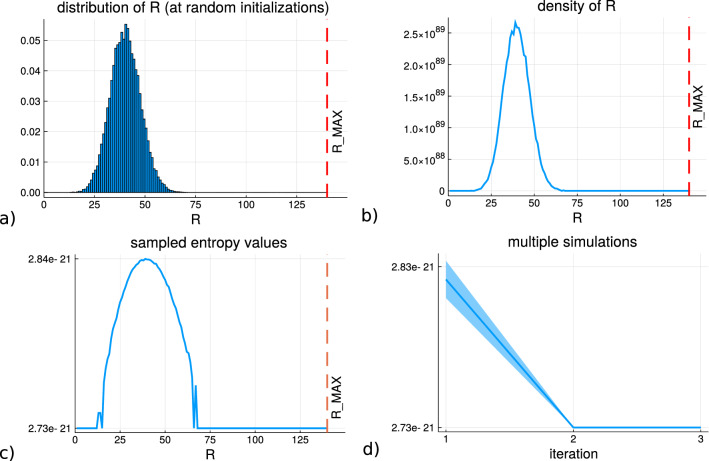
The plots here show how the progression of the Schelling model produces a decrease in entropy with the iterations (which is introduced in^25^). **(a)** shows the distribution of the grid homogeneity value *R* across all the agents on the grid at random initializations. Considering *R* as a macrostate, **(b)** plots the density of the macrostate across different values $\Omega (R)$. **(c)** shows the entropy values $S_R$ for the macrostate values, and in **(a)**–**(c)** the red dashed line is the largest value *R* can take for the number of agents on the grid. **(d) ** shows the trace of the entropy calculation across multiple simulations and the standard deviation of the values.

What is a common feature of previous work in sociophysics for this modeling development is that the progression of the Schelling model states are monitored by looking at the percentage of the agents which have achieved the homogeneity satisfaction or the related measure of energy of the system due to the local interactions over the grid. An alternative approach is taken in^25^ where the entropy over the simulation trace is estimated. It provides an equation set for the Schelling model in which the individual configurations of the agents within the grid at any time point can be considered as a microstate, $\mathbf {r}_t$, belonging to a macrostate at each time point, $R_t$ which is the overall number of satisfied residential agents. The density of the macrostates, $\Omega (R)$ is estimated so that the entropy can be calculated $S_R = k_B\text {ln}\Omega (R)$ for each macrostate value that the microstates belong to. The results of this study are summarized in Fig. 2. Subfigure (a) shows the distribution of the *R* macrostate values from random uniform samples of the grid permutations. Subfigure (b) shows the density of the macrostates, $\Omega (R)$, by factoring in the total number of possible permutations distributed according to (a) (using the de-labeling factor). Subfigure (c) shows the entropy values arising from each macrostate value. Subfigure (d) presents the mean entropy values and standard deviation over multiple simulations for each iteration (only the first 3 iterations are shown since there are no samples drawn from the larger macrostate values as the density is tightly focused upon the mode).

Looking at Fig. 2a it can be seen how Schelling model simulations will produce initial samples of grids with *R* in a specific region of large density, and as the dynamics move agents the decrease in the density will result in a decrease of the entropy as well. A decreasing trend on the grid can therefore be used as an alternative indicator that the residential agents are segregating over time (simulation iterations). From a more theoretical perspective, it can be appreciated that this simple model dynamic is capable of taking the state of the agents into a macroscopic state which is very unlikely to occur by random and that it is consistent in its ability to achieve this decrease. Interesting as it may be, there is another possibly more important use case to be considered here in that the initial samples which the agents are randomly placed on the grid do not produce $R=0$ or values close to the lower bound. As noted in^26^, and later in^27^, there is a common misconception in the interpretation of entropy that configurations of high entropy would produce no clustering (adjacency of agents). From Fig. 1a it can be seen that the initialization produces some regions with clustering of homogeneous agents but that this did not occur with any of the Schelling segregation dynamics to begin with. A state of the grid where the labels assigned do not act according to the Schelling dynamics produces a high level of entropy irrespective when movements are made if agnostic of the labels. Therefore in order to determine whether the residential agents are moving independent of the labels the homogeneity minimization would require a reduction in the entropy and an actual awareness of the label in contrast to the maximization of the entropy measure which intrinsically allows for sporadic clustering (which could be considered false positives for segregation intentions if a minimization upon *R* is used).

An issue with the current approach to the Schelling model is that the system moves the agents in a direction for a decrease in entropy of the grid state which is not in line with the desire to have physical analogies of the system dynamics that respects the *arrow of time*^28^. The increase of entropy in physical systems over time, as stated in the second law of thermodynamics, is applied as a key concept in the modeling of global climate as the ’maximum entropy production principle’^29^ and in ecological system models^30,31^. This principle can be applied ubiquitously to most system models^32,33^ and those which do not produce an increase in entropy are said to introduce a ‘paradox’^34^. This paradox displayed in multi-agent systems has been noted in studies of other social system models^35^ and although they can accurately capture the system dynamics and provide competitive predictions there is room to provide a more complete physical analogy.

The model proposed here to alleviate this issue introduces a monetary variable which is a property of the agents that does not influence the moves, but is altered by the movement choices. By having this monetary variable, the expenditure incurred by movements results in a dispersal of value creating a trajectory of the states of the system in two dimensions. In order to introduce a monetary variable into the Schelling model, each resident is allocated a sample from the distribution of the 2014 USA social security administration income reports (following the motivation of^36^ to use income data with justifications discussed in the Supplemental Information). As residential agents change positions in the grid they subtract a portion of the monetary value they have and evenly distribute it between the adjacent agents of the new cell they will occupy which updates the monetary variable representing income over the simulation. It is assumed that the movement incurs a relocation cost which operates as a type of ‘friction’^37^ decreasing the monetary store of the moving agent while its new neighbors absorb this loss of value by being able to offer services and from the rise in their current property values due to investment^38^. There are income brackets^39^ which can be found for the agents and if the difference in the monetary variable spans fewer than a predefined number of income brackets then those agents are considered to have the monetary homophily as considered for the labels. For an agent the number of adjacent agents which satisfy the income bracket homophily will determine whether the agent is satisfied in the same manner as with the Schelling model in respect to the group label. The grid state will use the aggregate of these satisfactions. An initial income allocation based upon a uniform income process will also be used for comparison. The density of the states is estimated from samples of the grid microstates using both the label and income satisfactions so that entropy values for a trajectory can be allocated. Details are provided in the Supplemental Information regarding the data used and the income allocations while the Methodology section will discuss in more detail the dynamics. This introduced dimension is another macrostate, *I*, the model trajectory then produces a macrostate value pair (*R*, *I*), and the density of the space for the residential and the income homogeneity, $\Omega (R,I)$. It will be used so that the entropy for the macrostate variables $S_{R,I} = k_B\text {ln}\Omega (R,I)$ can be found.

The importance of considering the entropy trace of the Schelling model is stated in the work of^40^ where the authors argue that the transformations of the grid states could be caused by ’entropic’ effects and since those results presented are based upon ratios of agent population features it leaves the task of computing the entropy trace as a worthy pursuit to complete. From a more general perspective for systems theory^41^, poses the question of “does the second law of thermodynamics apply to social systems or not?” and the Schelling model can help in answering this question if the model captures more key variables. In^42^ a description can be found for the scope of using entropy in general for sociological modeling. Reference^43^ describes how economic factors can be reasoned about with entropy and their relation to econophysics.

## Methodology

The outline of the methodology will follow a similar notation set used in^25^. The grid in which residents are placed and allowed to move within will be represented by a lattice $\Lambda$ of $d=2$. Each position within $\Lambda$ is referred to as a cell of which there are a total of $N = |\Lambda |$ and are indexed linearly via $n \in [1,\ldots ,N]$. The square lattice will have $N=192$ and 70 agents for each group so that the maximum satisfaction for the grid is 140 (each agent of each group label having a satisfied homogeneity). In this study there are 2 type of residents and three types of cell memberships, $m_n \in \{m_{group1},m_{group2},m_{empty}\}$. The residential macrostate variable that is the number of agents on the grid which have their local homogeneity satisfied is denoted by *R*, and the grid total number of agents surrounded by other agents with income brackets (as described in the Data section of the Supplemental Information) close to its own, is the monetary macrostate variable *I*. The density of the space for these variables is estimated through sampling and the entropy calculations on this space (*R*, *I*) is produced.

Linear indices are used to map to the grid coordinates, $m_n \Longleftrightarrow m_{(ni,nj)}$, which indexes the cells $1,\ldots ,N$. The calculation for an agent’s local homogeneity total is found from:

1 $$\begin{aligned} l(m_n) = \sum _{i=-1}^{1}\sum _{j=-1}^{1} \left( \delta _{m_{(ni,nj)},m_{(ni+i,nj+j)}} : i,j \ne 0 \right) . \end{aligned}$$

where the $\delta$ is the Kronecker delta. The local homogeneity value is used to evaluate the criteria for the agent homogeneity satisfaction via:

2 $$\begin{aligned} r\left( m_n\right) = {\left\{ \begin{array}{ll} \left( l\left( m_n\right) \ge h \right) &{} \, \text {if} \, m_n \notin \left\{ m_{empty} \right\} \\ 0 &{} \, \text {if} \, m_n \in \left\{ m_{empty} \right\} \end{array}\right. }. \end{aligned}$$

The cells not on an edge require four similar cells adjacent to it ($h=4$), cells on an edge but not a corner need three ($h=3$), and the cells in a corner require two equally labelled cells ($h=2$) or more to be satisfied on this constraint. These values will be binary for each cell and the grid is stored as an ordered set of agents for each iteration *t*:

3 $$\begin{aligned} \mathbf {r}_t = [r(m_1,t),\ldots ,r(m_N,t)]. \end{aligned}$$

The macrostate summation over the whole grid using linear indices is found via $R = \sum ^N_{n=1}r(m_n)$ and using the time index:

4 $$\begin{aligned} R_t = \sum ^N_{n=1}r(m_{n,t}). \end{aligned}$$

The number of microstates (the density) for a particular macrostate value *R* is represented as $\Omega (R)$, and over the iterations each microstate belongs to one of the macrostates, $\mathbf {r}_t \in R$. It is considered that each microstate is drawn from the density *R* with the same probability, $p(\mathbf {r}) = \frac{1}{\Omega (R)}$. The entropy of the macrostate is denoted by $S_R = k_B\text {ln}\Omega (R)$. As the simulation progresses the macrostate value at each iteration can be used for the entropy trace ($R_t$);

5 $$\begin{aligned} S_t = k_B\text {ln}\Omega (R_t). \end{aligned}$$

The distribution of $\Omega (R)$ is sampled with Monte Carlo where the $\mathbf {r}$ microstates are drawn uniformly $\mathcal {U}(\mathbf {r})$. The de-labeling factor is used for the total number of permutations of the agents on the grid, $\frac{N!}{(\prod ^{group}_g N_g!)}$ where $N_g$ is the number of agents of each group, so that the distribution of the macrostate samples can be used to partition the expected number of microstates of each macrostate. The relationship for the total macrostate space is found with:

6 $$\begin{aligned} \sum ^{R_{max}}_{R=R_{min}}\Omega (R) = \frac{N!}{\prod ^{\Vert group\Vert }_{g=1}N_g!}. \end{aligned}$$

Using the probability from the sample numbers for each macrostate, *p*(*R*), the sampled density of each macrostate can be found;

7 $$\begin{aligned} \widetilde{\Omega (R)} = p\left( R\right) \times \frac{N!}{\Pi _{g=1}^{ \Vert group \Vert }N_g!}, \end{aligned}$$

and the probability for a macrostate is calculated $p(R) = \frac{\Vert \mathbf {r}\in R\Vert }{K}$.

The monetary variable is produced from income data and referred to as income in the model. These values are sampled from real data (USA social security incomes) and from a uniform distribution of income allocation. Each cell in the grid $m_n$, which is not empty, is assigned an income bracket, $m_{n_b}$. The income brackets are used to assess similarity between the agents as noted in the Data section of the Supplemental Information. $b \leftarrow f_b(i)$ produces a bracket number for each income value, and $m_{n_b} \Longleftrightarrow m_{(ni,nj,b)}$ converts the linear indices back to grid coordinates. The income of each agent is denoted with $m_{n_i}$. The analogue of Eq. (1) of the local label homogeneity for the monetary variable using the income brackets is:

8 $$\begin{aligned} l_b(m_{n_b}) = \sum _{i=-1}^{1}\sum _{j=-1}^{1} \left( \left( | m_{(ni,nj,b)} - m_{(ni+i,nj+j,b)} | < h_{b_1} \right) : i,j \ne 0 \right) . \end{aligned}$$

Here $h_{b_1}$ is the threshold for the maximum difference in the number of income brackets between neighbors^36^ for homogeneity to exist and is set to $h_{b_1} = 4$. As Eq. (2), shows how the satisfaction of the Schelling constraint is computed for the group label association, the income bracket similarity association is found with:

9 $$\begin{aligned} b_h(m_{n_b}) = {\left\{ \begin{array}{ll} \left( l_b\left( m_{n_b}\right) \ge h_{b_2} \right) &{} \, \text {if} \, m_n \notin \left\{ m_{empty} \right\} \\ 0 &{} \, \text {if} \, m_n \in \left\{ m_{empty} \right\} \end{array}\right. }. \end{aligned}$$

As with the label associations, the $h_{b_2} = 4$ (agents not on an edge), $h_{b_2} = 3$ (on an edge but not corner), and $h_{b_2} = 2$ (on a corner). Each grid produces a set of income bracket homogeneity satisfactions which is a binary vector:

10 $$\begin{aligned} \mathbf {b}_t = [b_h(m_{1_b,t}),\ldots ,b_h(m_{N_b,t})]. \end{aligned}$$

The macrostate variable *I* for the income is found from:

11 $$\begin{aligned} I = \sum ^N_{n=1} b_h(m_{n_b}). \end{aligned}$$

For Schelling model simulations which use the real data distribution, $m_{n_b}$, is sampled using the CDF of reported incomes. In the Supplemental Information section ’Uniform Income allocation’ the algorithm for the generation of the uniform incomes is provided and figures for the distribution of the incomes across the agents.

**Figure 3:**
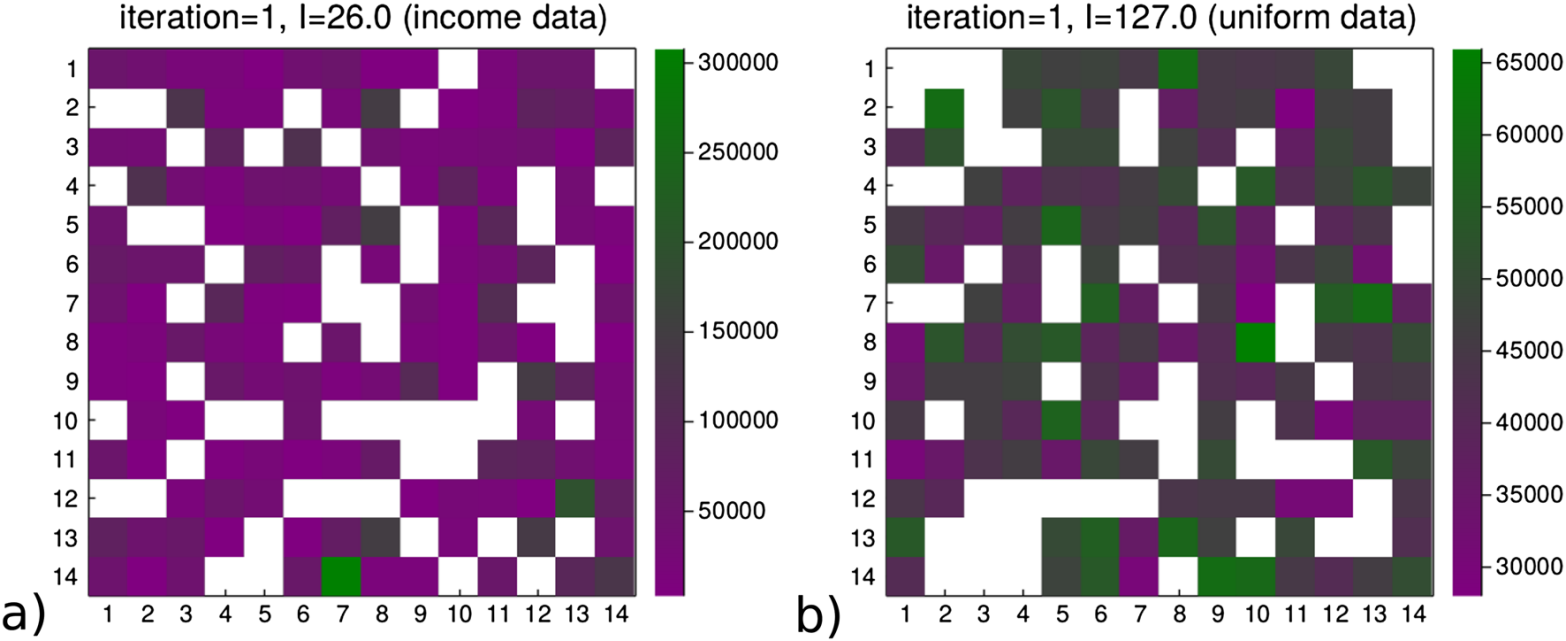
Examples of the monetary variable applied to the agents on the grid. **(a)** shows an initialization of a Schelling grid where each agent is allocated an income independently from the distribution of incomes reported by the USA social security administration, and the local income homogeneity for the agents across the grid is $I=26$. **(b)** shows the initialization of another Schelling grid where the monetary variable sample for each agent comes a process of uniformly allocating portions of a total income across the agents, and the income homogeneity for the agents on the grid is $I=127$.

Figure 3 displays examples of the income grids produced by the 2 different allocation processes. Subfigure (a) shows a grid where the incomes of the agents is sampled by the data distribution shown in the Supplemental Information Data section coming from real income data. Subfigure (b) shows the income allocation using the uniform process where there are fewer outliers and a smaller range of values. The income data macrostate value in (a) is less than that in (b), $I=26$ compared to $I=127$.

The monetary variable of grids can be initialized with $M_{n_i}$, coming from income data, or $M_{n_u}$ from the uniform allocation using the same total. Along with the sample of the agent positions within the grid, the vectors $\mathbf {r}$ and $\mathbf {b}$ for the initialization ($t=1$) allows the density of the macrostates to be estimated in a manner similar to Eq. (7). The same de-labelling factor $\frac{N!}{(\prod ^{group}_g N_g!)}$ is used in conjunction with the n-tuples for the income components. The sample probability for the grids is $p(R,I) = \frac{\Vert \mathbf {r,b} \in \mathbf {R,I} \Vert }{K}$ for *K* samples is used,

12 $$\begin{aligned} \widetilde{\Omega (R,I)} = p(R,I) \times \left( \frac{N!}{(\prod ^{group}_g N_g!)} \times \left( 70^ \frac{\sum \mathbf {m_{n_i}}}{1000} \right) ^2 \right) . \end{aligned}$$

The density at the macrostate of the grid can then be used to find the entropy for each macrostate value the microstate configuration is in:

13 $$\begin{aligned} S_{R,I} = k_B\text {ln}\widetilde{\Omega (R,I)}, \end{aligned}$$

which permits the estimation of the entropy for a trace of a simulation; $S_{R,I,t} = k_B\text {ln}\widetilde{\Omega \left( (R,I) \right) }_t$.

**Figure 4:**
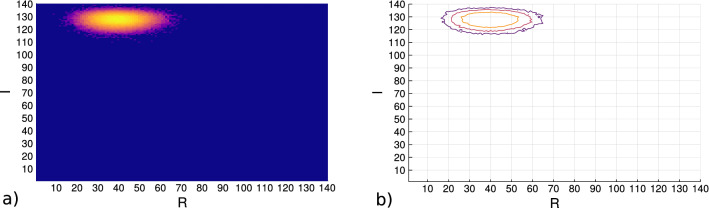
The sampled entropy values for the macrostate variables *R*, *I* for the proposed Schelling with the monetary variable. **(a)** shows the heatmap of the values and **(b)** the contours of the values sampled. The spatial positions of the agents are randomly allocated and the incomes for the agents come from the uniform allocation process.

Figure 4 shows the entropy contours across the domain of (*R*, *I*). These samples come from a uniform allocation of the residential group labels and the process of the uniform income allocation. Subfigure (a) displays a heatmap and Subfigure (b) the contour map for the changes in the values across the macrostate values (100,000 samples were used and further increases in the sample number did not substantially increase the range of the samples from the center of the density).

At each move of the Schelling algorithm, the proposed model here conducts an operation which captures a basic effect of expenditure for the move that is absorbed by the surrounding local agents. A 5% is subtracted from the moving agent’s income variable for each new adjacent neighbor which is then randomly distributed in that neighborhood. This dynamic is outlined in Algorithm 1. This is included in the Schelling simulations where the income variable information is used. Also a variant of the Schelling algorithm may be employed where the agents search for new empty cells to occupy if the destination provides label homogeneity (regardless if there is satisfaction already). The 5% can be substituted with other values to observe the effect and in this study it was found that 5% produces a similar number of steps for both the residential and income dynamics to reach the plateau from initialization.

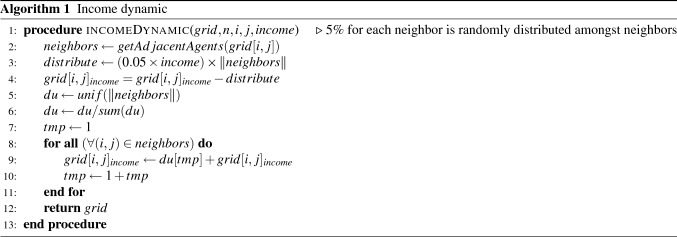



## Results

**Figure 5:**
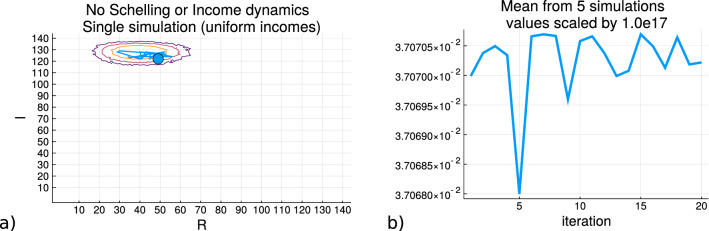
Example of the entropy trajectory under a simulation with random grid movements, no income dynamics and the incomes are obtained from a uniform allocation. **(a)** shows the values of the macrostates the simulation passes through in relation to the value of the entropy each state is sampled to have and the blue circle represents the initial starting point. **(b)** shows the mean value of the entropy trace of 5 independent simulations.

Figure 5 shows the results of running a simulation with random movements and no income dynamics when the initial starting position is produced with incomes coming from the process of uniform allocation. The random spatial arrangements of the agents is analogous to what is shown in Subfigure (a) of Fig. 1, but the movement patterns are not governed here by the Schelling criteria where a local homogeneity constraint is satisfied but random movements between empty cells. The incomes for the agents on the grid will resemble that shown in Subfigure (b) of Fig. 3. Here Subfigure (a) shows the trajectory of a single simulation where the blue circle is the starting point and the contours shows the regions of the entropy values depicting the changes in the values over the regions. It can be seen how the trajectory remains in the areas of the largest entropy values. Subfigure (b) shows the entropy value trace over five independent simulations and that the values can display various changes in values as they cross between regions of the contours within *R*, *I*. This shows that spatial rearrangements themselves do not produce repositioning outside of the regions of largest sample densities which produce the largest entropy region. As shown in Eq. (13), the entropy trace is produced from $S_{R,I}$.

**Figure 6:**
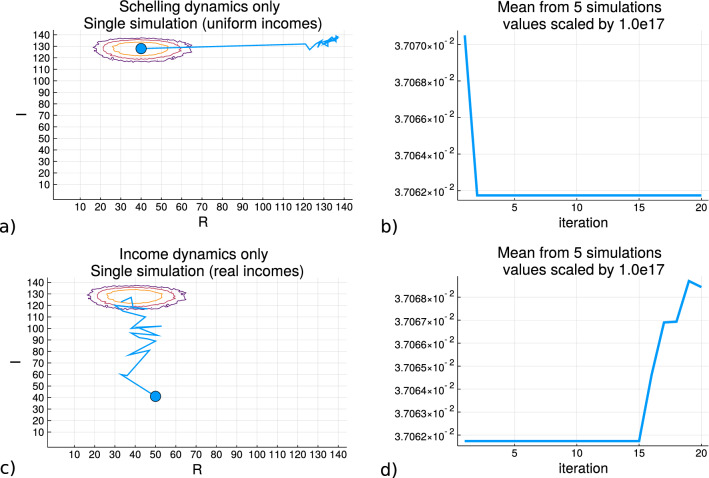
The entropy traces for simulation involving either only the spatial Schelling dynamics or only the income dynamics. **(a,b)** come from the model where the Schelling dynamics operate upon the agents whose income values are allocated from the uniform process and the entropy of the trace is shown which decreases as the value of *R* (for homogeneity) increases. **(c,d)** are produced from the model where the spatial movements are random placements and the incomes are allocated from the real data distribution from the USA social security data. There is an increase in the entropy as the state enters the region of greater sample density of the macrostate.

Figure 6 shows the results of running simulations with only the residential dynamics for *R* (without income dynamics), and the results of the simulations with only income dynamics operating on *I* (without the Schelling criteria). Subfigure (a) shows the results of initializing a grid where the agents are allocated incomes from the process of uniform income allocation rather than sampling it from the real data distribution, and the Schelling dynamics are applied to the state of the grid (shown in Supplemental Information section ’Uniform Income allocation’). At each iteration the simulation moves agents so that they achieve a local homogeneity satisfaction from their neighbors where the starting point is indicated by the blue circle. Although there are movements there are no changes to each agents’ income variable values due to these spatial rearrangements. The heatmap of the contour lines is shown as presented in Fig. 4. Subfigure (b) shows the mean trace of the entropy values along the simulation iterations of (a) for five simulations, and how it decreases as the state of the agent arrangments on the grid corresponds to regions of lower entropy on (*R*, *I*). Subfigure (c) shows the trace of a simulation where the grid initialization allocates the monetary variable *I* according the real income data distribution shown in the Supplementary Figure in the Data section, and there are random spatial movements with income dynamics applied. As described in the Methodology section, the income dynamics subtract 5% of an agent’s monetary value for each new neighbor at the destination cell and distributes this total randomly between these new neighbors of the agent. Subfigure (d) plots the trace of the entropy values across five simulations and the increase can be seen when the path enters the region of the contours.

The results of Subfigures (a) and (b) mirror what is shown in Figs.  1 and 2, with the added component of the monetary variable for each agent is presented, as depicted in Fig. 3 (subfigure b) since that grid has the incomes allocated according to the uniform process). Since the incomes are allocated from a process of uniform allocation, there is less skew and variance so there are more agents with similar incomes producing a higher value of *I* than with the real data distribution. Since the differences are typically not large enough in the income variable for each agent for movements to change the monetary local homogeneity, *I* stays relatively the same over the iterations. The Schelling criteria is applied for the movements so that as the samples initially are drawn from the region of largest density in *R*, *I*, the trajectory towards larger *R* decreases the entropy values as the distance from the dense contour center increases.

Subfigures (c) and (d) have incomes sampled from the real data distribution of incomes reported by the USA social security administration shown in the Supplementary Information section ’Uniform Income allocation’, where there is larger variance and skew so that a grid with agents sampling incomes from that distribution will have a smaller aggregated income homogeneity *I*. Although the movements are random placements without the Schelling criteria, the income exchanges between the agents results in an increase of the local income homogeneity values of the agents and therefore the overall grid macrostate value *I*. This change over the simulation iteration results in the movement towards the region of the largest sample density with increased entropy values since the *R* value came from the largest region to begin with and that random movements to not change it substantially. Two important points to note is that the number of iterations required for the income dynamics to produce a change in $S_{R,I}$ is larger than for the Schelling dynamics altering the label homogeneity, and that the income dynamics can effectively take the state of the grid from a low entropy region into a high entropy region. It is not necessary that the scale of the macrostate value changes are equal as can be seen by the shape of the entropy value contours.

### Continuous homogeneity movement Schelling model with the income dynamic applied

**Figure 7:**
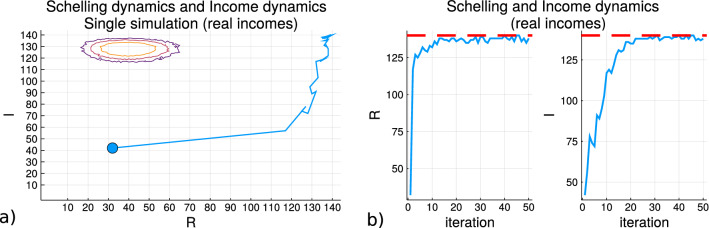
The simulations using the Schelling model with income dynamics when continuous movements are applied. The continuous movement here means that agents continue to change positions in the grid even if homogeneity satisfaction is achieved if destination position also results in satisfaction. **(a)** shows the value of the macrostate variables and **(b)** shows the trace on the entropy contour plot where the blue circle is the starting point.

Figure 7 shows the results of running simulations with the continuous Schelling model which is a modification to the classic model. The modification is that residential agents can continue to move between grid cells while still maintaining local label homogeneity satisfactions instead of remaining in the same cell if the homogeneity criteria is satisfied. Subfigure (a) shows the trace of a simulation for the values of the macrostate variables *R*, *I*. The blue circle is the starting point of the initialization and the contours are of the entropy values from the density samples $S_{R,I}$. Subfigure (b) shows the individual macrostate variable value progressions over the simulation. It can be seen in both plots that the simulation stays within the highest possible values of both variables with minor oscillations.

As a result of the continuous movements it can be seen that the *R* values are not monotonic increasing or producing a flat line after a certain point because of the inter-dependency between agents for satisfying the homogeneity criteria. This can be compensated for in subsequent iterations and it is why there are oscillations seen for *R* but were not observed previously. The continuous movements also ensures that the value of *I* continues to increase with the increased number of movements because each move results in the income spread. The *R* values increase towards the maximum at a faster rate than *I* does and if the movements ceased when *R* approached the maximum the progression of *I* would be halted. So it can be seen that an increased number of spatial movements are needed in order to increase both macrostate variables to the same levels. This implies agents may balance their movement patterns in order to retain their monetary differential by refraining to move unless a constraint chosen must be satisfied. The path taken in $S_{R,I}$ increases both macrostate variables which from the previous simulations shown in Fig. 6 decreases the entropy in respect to *R* but increases it in respect to *I*.

### Schelling model simulation with the income dynamic

**Figure 8:**
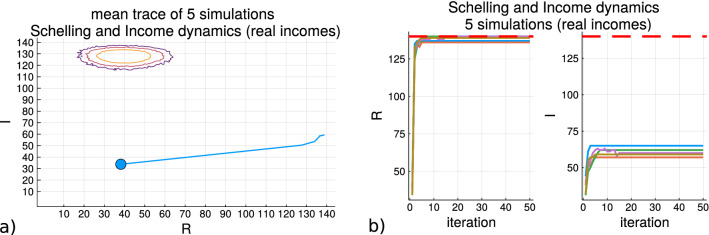
The Schelling model with the monetary variable simulation results when the income for the agents is sampled from the distribution reported from the USA social security administration. In **(a)** the macrostate variable traces for the simulation are shown. **(b)** shows the mean trajectory of 5 simulations where the blue circle is the average starting point.

Figure 8 shows the results of the Schelling model simulation with the monetary variable *I* included. In this simulation the income distribution of the agents is sampled from the real data obtained from the USA social security administration, and is then normalized so that the total accumulated sum of incomes is constant across all the simulations presented. Subfigure (a) shows the mean trace of five simulations where the coordinates are the values of the (*R*, *I*) macrostate variables. The blue circle places the average coordinate of the starting random initialization point, and the contours are produced from the sampling of the entropy values $S_{R,I}$ in this space as shown in Fig. 4. Subfigure (b) shows the traces of the two macrostate variables over the iterations of the five simulations. The results of this subfigure shows that although the original context of the Schelling model is preserved with the effective quick increase in *R* to the maximum value or close to it across all the simulations, the local homogeneity of the *I* variable does not see an such an increase. After the spatial movements cease to be made the income dynamics also do not affect the agents and the corresponding flat trace lines can be seen.

In Fig. 7 the results do not show the flat traces indicative of a convergence since that modified Schelling model allows for the agents to move at each iteration as long as there is spatial satisfaction achieved, and why there are oscillations close to the maximum values of both macrostate variables. It could be expected that the increase in the spatial homogeneity variable should be compensated by an equivalent change in the income homogeneity as the spatial variable moves away from the high density region as much as possible on *R*, but the income variable has a smaller change on the scale of *I*. These scales though do not have to be equivalent as can be seen by the non-circular shape in the contours of the entropy samples. The contours of the entropy density values show that the changes are on an ellipse so that there is a faster rate of decay from the center for *I* than *R* because between the maximum and minimum values of *I*. From Fig. 6 it can be seen how the direction of change in *R* produce here supports a decrease in the entropy, while the increase in *I* supports an increase.

## Discussion

The Schelling model of segregation provides a insight to how populations change their residential positions based upon identity label homophily. The trace of the simulations with this models shows a decreasing entropy trace which goes against the maximum entropy principle if it is to find a correspondence with models in physics. In order to address the issue of a decreasing entropy trace in the Schelling model, another variable is introduced which operates within the Schelling model but without disrupting its original formulation. A monetary variable can be introduced as attributes of the residential agents with a dynamic for the changes incurred from movements. This monetary variable for the residential agents in the model is sampled from government reported income data as is done in^36^. The aggregate homogeneity values for the residential component among the agents and the income component allow a simulation with to produce an entropy trace accounting for both variables (Eq. (13)). As a result it can be seen that as the residential dynamic works to decrease the entropy the income dynamics bring the states closer to higher density regions (Fig. 4).

The results show how that the real income data sampled for the agents produces lower aggregate income homogeneity values than when the incomes are sampled uniformly for the initialization of a grid, and brings the entropy for those real income sampled states to lower values. Simulations with real income data operating without the Schelling residential homogeneity (random grid movements) show that the entropy trace increases as the income dynamics upon movements induce larger income homogeneity values, while the residential homogeneity values stay roughly within the initialization region of high entropy (Fig. 6). Figure 6 demonstrates that the model captures an effect which balances the reduction in the entropy due to the residential homogeneity with state changes in the direction of an increase of entropy due to the initial income skews being reduced from movement expenditures. By introducing the monetary component into the Schelling model dynamics the simulation then supports a key principal in physics, the second law of thermodynamics, that systems progress towards an increase in entropy.

In order to create an increasing entropy simulation trace an alternative modification could be the introduction of multiple identity labels for the agents. A ranked order upon the satisfaction provided by residing in homogeneous regions of different labels would produce different values of entropy considering the labels independently and a swapping of the rank order of satisfaction provided by the different labels would incur a wide range of movements which would lead to a new configuration where there would be increases and decreases during these periods of reconfiguration. The changes would only be visible while the states move in order to reach a new overall stability at the same entropy value. This could be an interesting avenue for future work.

All the code used in this study was written in *Julia*^44^ (using version 1.4), within a single Jupyter notebook^45,46^, and the implementation will be available on Github under the account at https://github.com/mantzaris.

## Supplementary information

Supplementary Information.

## Data Availability

The software will be made available as a repository under the Github account https://github.com/mantzaris, as a single Jupyter notebook for the Julia Lang kernel developed on version 1.4.
