# Supplementary material for: Incorporating a monetary variable into the Schelling model addresses the issue of a decreasing entropy trace
Source: Sci Rep. 2020 Oct 12;10:17005. doi: 10.1038/s41598-020-74125-6 (PMC7552411; doi:10.1038/s41598-020-74125-6)
Supplement: Supplementary file 1 — Supplementary Information. [file 41598_2020_74125_MOESM1_ESM.pdf]

Incorporating a monetary variable into the Schelling model addresses the issue of a decreasing entropy trace

Alexander V. Mantzaris[1,*]

[1]University of Central Florida, Department of Statistics and Data Science, Orlando, 32816, USA
[*]alexander.mantzaris@ucf.edu

ABSTRACT

This provides supplementary information to the main text which motivates the development of a Schelling model with a monetary variable in which the entropy of the traces can be estimated. The information provided here gives more insight into the data used on allocating the incomes and the generation process for the uniform income allocations.

Data

The work of[1] presents a study of the Schelling model applied to urban regions of Israel where income data is incorporated into the model to explain the spatial arrangements (more detailed view provided in[2]). The work does discuss 'wealth' of neighbors as an important component of the decision making of households when changing locations, and that income data is an accessible information source for the researcher looking to incorporate the monetary variable into models such as Schelling. Wealth is a more general monetary term which can include assets, but income is used since it is related to taxable quantities the government uses and information on it can be found from open data sources more easily as shown in the Figure 6 of[1]. The data used in this exploration comes from the 2014 USA social security administration report which is available at `https://www.ssa.gov/cgi-bin/netcomp.cgi?year=2014`, and can be found in a more readily usable format (CSV) from `https://raw.githubusercontent.com/jkeesh/which-percent/master/data.csv`. Figure 1 shows the empirical CDF of the distribution for the incomes of the USA working population given this data. From this can be found the function $\hat{F}(i)$ which gives the fraction of the population which have an income i or less. Provided with this data is also a list for the income bracket membership[3] of each income i which is ordered $b \leftarrow f_b(i)$. These brackets are provided by the data source and are considered meaningful representations for differentiating different income earners. Instead of direct amount differences the number of income brackets are used to examine income similarity. It is of particular interest to observe the lack of symmetry about the mode and its skew is noted from an earlier study from a larger set of years in[4]. This work of[4] interestingly also states that the upper end of the distribution are have large values 'earning'/'wealth'/'income' together while these 3 dimensions do not correlate in the same way for the lower end of the distribution.

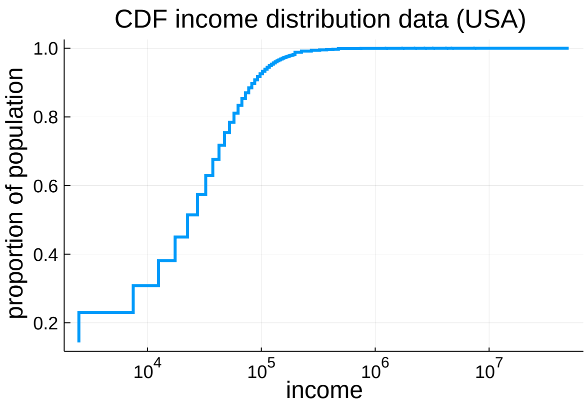

Figure 1. The CDF of incomes for USA citizens from the *social security administration*.

Algorithm 1 Uniform income allocation

procedure UNIFORMINCOMEALOC($N, alocSize, \mathbf{m_{n_i}}$) $\triangleright$ allocate the total amount to agents uniformly
  $total \leftarrow \sum \mathbf{m_{n_i}}$
  while $total - alocSize \nless 0$ **do**
    $n \leftarrow unif(1, N : n \notin m_{empty})$
    $m_{n_u} \leftarrow m_{n_u} + alocSize$
    $total \leftarrow (total - alocSize)$
  end while
  $n \leftarrow unif(1, N : n \notin m_{empty})$
  $m_{n_u} \leftarrow m_{n_u} + total$
  return m_u
end procedure

Uniform Income allocation

The density of the sample space for the macrostate values of I are found by uniformly sampling the distribution of the microstates of the income upon a uniform allocation process. This is outlined in Algorithm 1 where the income of each agent at initialization is denoted with m_{n_u} (m_{n_i} refers to the incomes sampled from the income data). The total sum of the sampled income data is used so that both samples have the same total across the grid. In these samples $alocSize = 1000$ to make the allocation procedure faster. Figure 2 demonstrates the uniform allocation process results. Subfigure a) shows the distribution of the incomes across the agents for a single sample grid. Subfigure b) shows the distribution of I (which is the macrostate for the grid income bracket homogeneity) across different independently uniformly allocated grids. It can be noticed how the values are close to the maximum possible 'I MAX'.

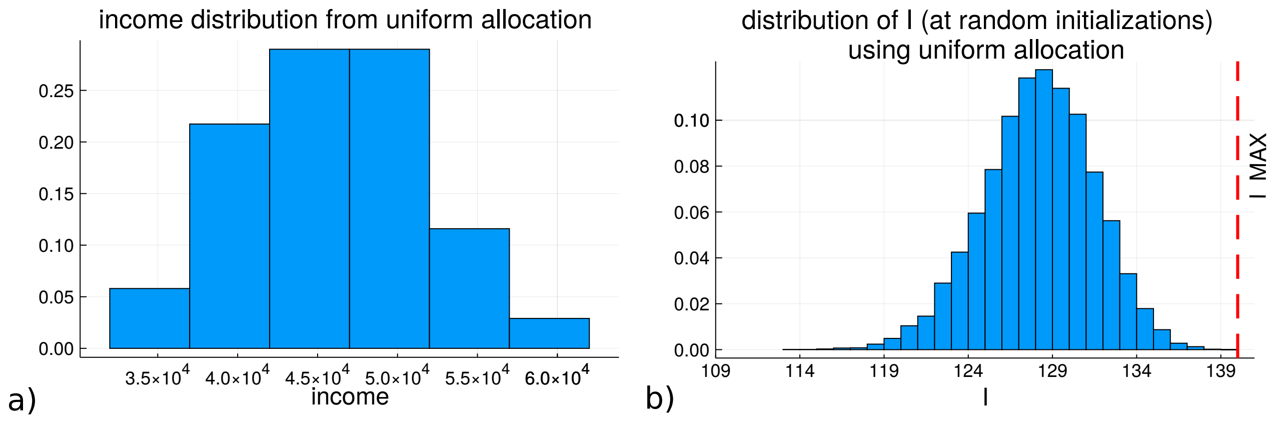

Figure 2. The state of the grid when agents are allocated monetary values from a real income distribution is compared to the state when the same total amount is uniformly allocated. Subfigure a) an example of the distribution of income values across the agents when the uniform allocation is applied. Subfigure b) shows the distribution of the income homogeneity value I from random initializations of the grid when the process for uniform income allocation is applied. The dashed red line indicates the largest income homogeneity obtainable for the grid.

References

1. Benenson, I., Hatna, E. & Or, E. From schelling to spatially explicit modeling of urban ethnic and economic residential dynamics. *Sociol. Methods & Res.* **37**, 463–497 (2009).
2. Hatna, E. & Benenson, I. The schelling model of ethnic residential dynamics: Beyond the integrated-segregated dichotomy of patterns. *J. Artif. Soc. Soc. Simul.* **15**, 6 (2012).
3. Scarboro, M. State individual income tax rates and brackets for 2018. *Tax Foundation* (2018).
4. Rodriguez, S. B., Díaz-Giménez, J., Quadrini, V., Ríos-Rull, J.-V. *et al.* Updated facts on the us distributions of earnings, income, and wealth. *Fed. Reserv. Bank Minneapolis Q. Rev.* **26** (2002).
